# Copy Number Variation of the *SOX6* Gene and Its Associations with Growth Traits in Ashidan Yak

**DOI:** 10.3390/ani12223074

**Published:** 2022-11-08

**Authors:** Xinyi Li, Chun Huang, Modian Liu, Rongfeng Dai, Xiaoyun Wu, Xiaoming Ma, Min Chu, Pengjia Bao, Jie Pei, Xian Guo, Ping Yan, Chunnian Liang

**Affiliations:** 1 Key Laboratory of Yak Breeding Engineering Gansu Province, Lanzhou Institute of Husbandry and Pharmaceutical Science, Chinese Academy of Agricultural Sciences, Lanzhou 730050, China; 2 Key Laboratory of Animal Genetics and Breeding on Tibetan Plateau, Ministry of Agriculture and Rural Affairs, Lanzhou 730050, China; 3 College of Life Science and Engineering, Northwest Minzu University, Lanzhou 730030, China

**Keywords:** CNV, *SOX6* gene, qPCR, gene expression, growth character, correlation analysis

## Abstract

**Simple Summary:**

The *SOX6* (sex determining region Y-box 6) gene belongs to one of the transcription factors in the SRY (sex-determining region Y) family, which affects sex determination, embryonic and nervous system development, bone and various organ formation. In the previous study, the whole-genome sequencing was used to detect multiple genes located in the copy number variation region, including *SOX6* gene. In this study, we identified the correlation between the growth traits and CNV of *SOX6* in 311 Ashidan yaks. The results showed that *SOX6*-CNV was significantly correlated with the chest girth of the 6-months old yaks (*p* < 0.05) and 30-months yaks (*p* < 0.05), and withers height of 6 months yaks (*p* < 0.05) and 18-months yaks (*p* < 0.05), suggesting the *SOX6*-CNV affect growth traits in yaks, and could be new markers for the selection of yak breeding.

**Abstract:**

Copy number variation (CNV) is a fundamental type of structural variation of the genome affecting the economic traits of livestock. The *SOX6* gene (sex-determining region Y-box 6), as a transcription factor, has multiple functions with regard to sex determination, embryonic growth, the nervous system development, as well as bone, and various organ formation. This study employed quantitative real-time fluorescence quota PCR (qPCR) for detecting the *SOX6*-CNV of the 311 Ashidan yaks and analyzed the correlation of the *SOX6*-CNV with four phenotypes (including body weight, withers height, body length, and chest girth) of the yaks aged 6, 12, 18, and 30 months using ANOVA and multiple comparisons. Furthermore, the *SOX6* gene expression was identified in seven different tissues of the yaks. The experiment results demonstrated the expression of *SOX6* in each tissue, and the kidney and muscle tissue were found to have higher relative expression levels. Based on the processing by IBM SPSS software, *SOX6*-CNV was significantly correlated with the chest girth of the 6-months old yaks (*p* < 0.05) and 30-months yaks (*p* < 0.05), and withers height of 6 months yaks (*p* < 0.05) and 18-months yaks (*p* < 0.05), as well as the normal type of CNV, was chosen for yak breeding. In conclusion, *SOX6* might be prominently involved in promoting growth and development of yaks, suggesting that the *SOX6* gene can be used in breeding yaks by molecular marker-assisted selection (MAS). The study also offered some important insights into the references and clues for the genetic breeding of yaks.

## 1. Introduction

Copy number variation (CNV) is defined as a DNA fragment with a variable copy number size of 1 KB or longer than the reference genome, which is a significant portion of the variant in the genome [1]. CNV mainly shows recombination, deletion, and insertion of multiple loci [2]. CNV is currently considered a polymorphic genetic marker of important economic traits or disease susceptibility phenotypic variation in livestock species [3,4]. Studies over the last couple of decades have shown CNV to be compactly related to important growth traits [5,6]. For example, the study conducted by Dorshorst has shown a complex recombination structure region on chromosome 20 of silky fowl, and the *END3* gene in this region was found to affect the excessive deposition of melanin [7]. The CNV of the *KIT* gene has been significantly associated with the dominant traits of coat color in pigs [8]. Another study on the Qinchuan cattle has reported the body height to be affected by the *GBP4* gene CNV [9]. Several literature have proved that CNVs are crucial for studying the differences in growth traits of domestic animal, providing an important reference value for breeding work.

Yak is an animal unique to the plateau region [10], that is mainly distributed in the cold plateau zone. The yaks in China account for more than 95% of the world. Yaks provide multiple resources such as meat, milk, wool, and transportation to the herdsmen [11]. This experiment used the Ashidan yak, which shows excellent production performance. Ashidan yak is hornless and therefore suitable for barn feeding breeding in the frigid plateau area. As a new breed, the Ashidan yak is beneficial for developing the ecology and economy of locals. So far, several studies have established that CNV can influence economic traits [12,13,14], and the application of CNV has great potential in livestock genetic breeding for improving the economic benefits.

The *SOX6* gene belonging to a transcription factor of the SRY (sex-determining region Y) family, was originally isolated from the testis of adult mice [15,16]. Over the last two decades, the literature has enlightened us about the *SOX6* gene, indicating that the *SOX6* gene might affect the nervous system [17], embryonic development [18], and sex determination [19]. In addition, the *SOX6* gene is also involved in regulating the specification of the muscle fiber type in mammals [20]. Studies on the effects of *SOX6* on the muscle and bone have identified the transcription factors in the SOX family to exert at point by targeting organs, showing interactions between the transcription factors. For instance, the proliferation and differentiation of the chondrocytes were regulated by the interaction of *SOX5* and *SOX6* with *SOX9* [21]. *SOX5* and *SOX6* were *SHOX* interacting proteins, and their interaction can affect bone formation and development [22]. Based on Northern blot technology, the *SOX6* gene was detected to be the highest in the skeletal muscle tissues of adult mice, indicating that *SOX6* might be involved in maintaining the muscles [23].

Our previous study has utilized the whole-genome sequencing technology for detecting multiple genes located in the CNV region, including the SOX6 gene [24]. These CNV regions were located in the quantitative trait locus (QTLs) and were closely related to the growth traits in yaks. So far, there has been no report on the CNV of the SOX6 gene in yaks. Therefore, based on preliminary sequencing data, we would like to explore the correlation between the SOX6-CNV and growth traits in Ashidan yak. Moreover, we expect it could provide data support for the genetic improvement of yak breeding.

## 2. Materials and Methods

### 2.1. Animal Welfare

The Lanzhou Institute of Husbandry and Pharmaceutical Sciences of CAAS (No. LIHPS-CAAS-2017-115) has approved all experiments in this study. The body size traits and the blood samples of the yaks were assessed according to the Guidelines for the Care and Use of Laboratory Animals.

### 2.2. Body Size Traits and the Blood Samples Collection

All the blood samples of 311 healthy female Ashidan yaks were collected from the Datong Cattle Farm, Qinghai Province, China; each blood sample was 4 ml. The body indices including body weight, withers height, body length, and chest girth were measured for 311 yaks aged 6, 12, 18, and 30 months, respectively. The method for the standard measurement can be based on the study by Gilbert [25]. Seven tissues of three 3-year-old yaks were sampled for analyzing the *SOX6* gene expression, for the tissues of the heart, liver, spleen, lungs, kidney, skeletal muscle, and adipose tissue. It is necessary to select yak raised under the same conditions and similar in body conformation. The three yaks were subjected to electric shock in this study to reduce the pain before death.

### 2.3. Isolation and Identification of Genomic the DNA and RNA

The genomic DNA was extracted from the blood using the EasyPure Blood Genomic DNA Kit. The RNA was isolated from the seven tissues of 3 yaks using the Trizol reagent and the RNA was purified using the RNase-free DNase based on the instructions. The purity and quality of DNA and RNA were determined using the Thermo Scientific NanoDrop 2000C and 1.2% agarose gel electrophoresis. Total RNAs were converted to cDNA using PrimeScript™ Reagent Kit and gDNA Eraser. DNA and cDNA samples were preserved at −20 °C. The main test reagent equipment and manufacturers in this experiment are shown in Table 1.

### 2.4. Information of Candidate Gene

**Figure 1:**
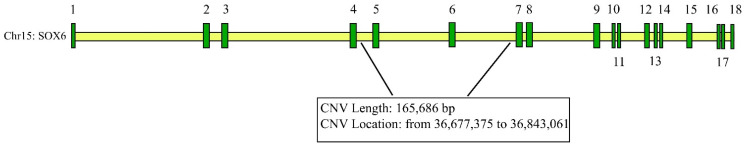
Information on CNVs of *SOX6* genes (the numbers from 1 to 18 denote the exons).

The CNV (chr15: 36,677,375 to 36,843,061) of SOX6 (AC_000172.1) is located in intron 4–6 (Figure 1).

### 2.5. Primer Design

The gene sequence was queried using National Center for Biotechnology Information (NCBI). The primers were designed by the Primer-BLAST online software for analyzing the CNV and gene expression. Meanwhile, the primers were designed according to general principles [26]. For CNV analysis, the primers should be within the range of the *SOX6*-CNV region, and the basic transcription factor 3 (*BTF3*), which is known to be a universal transcription factor 3 was the reference gene in this study. To design primers for analyzing the gene expression according to the mRNA sequence of the *SOX6* gene, the beta-actin (*β*-*Actin*) gene was selected as the reference gene. Details of the primer pair sequences information are shown below (Table 2). The primers and the optimal temperature of the primers were tested using the polymerase chain reaction (PCR) and 1% agarose gel electrophoresis.

### 2.6. Copy Number Variation Identification and Gene Expression

Studies of CNV have generally employed qPCR technology, which is known for its validity and convenience [27]. The qPCR was performed on the LightCycler^®^ 96 Instrument to detect the CNV-*SOX6*. A 20 μL reaction system was selected for the experiment, including 1 μL DNA/cDNA, 10 μL SYBR Premix Ex Taq II, 1 μL forward primers, 1 μL reverse primers, and 7 μL ddH_2_O. The qPCR procedure as shown below: 95 °C pre-denaturation for 30 s, 45 cycles involved 95 °C for 5 s, 59 °C for 30 s, after cycling, 5 s at 95 °C, 60 s at 65 °C, and finally at 95 °C continuously. All the experiments were replicated three times to ensure the accuracy of the experiment, and the final data were presented as mean ± standard deviation (SD).

### 2.7. CNV Correlation Analysis and Expression Profiling

The final value were calculated according to the formula:

2 × 2^−ΔΔCt^.

where ΔCt = Ct_target gene_ − Ct_reference gene_, ΔΔCt = ΔCt_test_ − ΔCt_control_, and all the analysis data were standardized [28]. The expression profiling of the *SOX6* gene was plotted using the GraphPad Prism 8.0 software. The relevance between the *SOX6*-CNV and four phenotypes was detected using the SPSS 26.0 software. The statistical method was ANOVA (analysis of variance) and the non-parametric test. Before ANOVA, the homogeneity test (Table S1) and normality test (Table S2) were carried out for each character in the different CNV regions. The general linear model approach was chosen considering the uncertain factors influencing the phenotypic value, including age, genetic effects, and environment. The correlation between the CNV and four growth traits was analyzed using the model:

Y*_j_* = *μ* + CNV*_j_* + e*_j_*.

where Y*_j_* represents the observed value of growth traits; CNV*_j_* represents the *SOX6*-CNV type effect; *μ* represents the total mean value of each character; e*_j_* means random residual and *j* represents *j*th CNV type.

## 3. Results

### 3.1. Expression Profiling of SOX6 Gene and Distribution of Different CNV Types in Yaks

**Figure 2:**
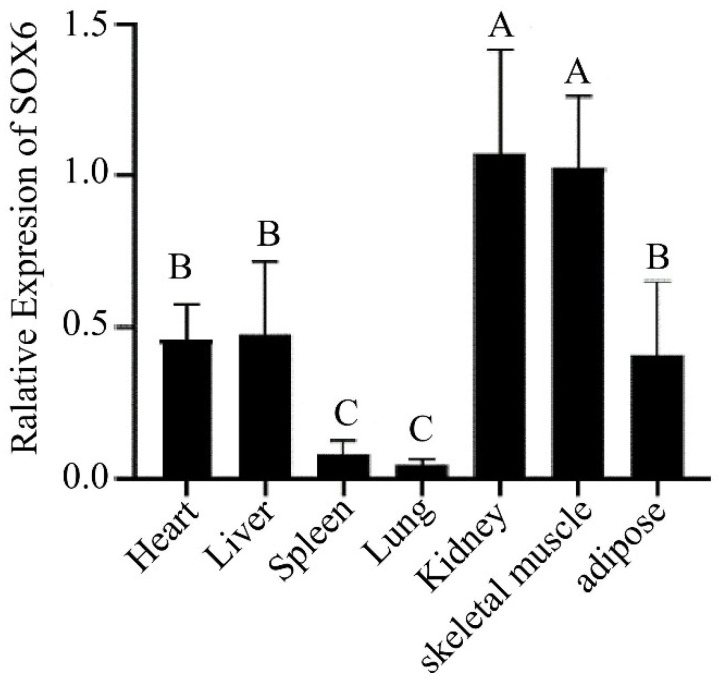
Tissues expression of *SOX6* in the Ashidan yak. Different capital letters (A, B, C) represent the extremely significant differences (*p* < 0.01).

The *SOX6* gene as a transcription factor regulates physiological functions such as regulating chondrogenesis, nervous system development, erythropoiesis, and other processes. The expression of the *SOX6* gene was detected in seven tissues of three yaks using qPCR, and the results are displayed in Figure 2. The *SOX6* gene was detected in all the tissues and the most abundant expression was found in the kidney and skeletal muscle tissues while low expressions were evident in the spleen and lung tissues. Furthermore, the experimental data were processed through the cycle threshold value for obtaining the type and frequency of the *SOX6*-CNV. The data were divided into three categories by 2 × 2^−ΔΔCt^ < 1 (Loss), 1 ≤ 2 × 2^−ΔΔCt^ ≤ 2 (Normal), 2 × 2^−ΔΔCt^ > 2 (Gain) [29]. The number of *SOX6*-CNV in Loss, Normal and Gain are 17, 182, and 112. By calculating the proportions of the three categories of data in 311 yaks, we found that the CNV distribution of *SOX6* gene was different among the three types of yaks. The loss type CNV frequency of the *SOX6* gene was found to be 5%, while that of the normal type CNV frequency was 59% and the gain type CNV frequency was 36%.

### 3.2. Correlation between the SOX6-CNV and Four Growth Traits at Different Ages of 311 Yaks

A multitude of studies has confirmed that CNV extensively affects the growth traits of animals. After the homogeneity of the variance test, the *SOX6*-CNV and growth traits were tested to verify the relative using ANOVA. The experimental evidence in Table 3 showed the correlation analysis between the different types of *SOX6*-CNV and four economic traits of Ashidan yak. The chest girth of 6-month yaks (*p* < 0.05), withers height of 6-month yaks (*p* < 0.05), withers height of 18-month yaks (*p* < 0.05), chest girth of 30-month yaks (*p* < 0.05) were significantly correlated with *SOX6*-CNV. Subsequently, multiple comparisons were made based on the ANOVA analysis of variance for evaluating the differences between the means. The different CNV types of *SOX6*-CNV were found to significantly affect the growth traits. However, the CNV advantage types of the withers height were not uniform, the loss type has a better withers height at 6 months of age but the advantage was not obvious. Moreover, the withers height of gain type was significantly superior to the other types at 18 months of age. Meanwhile, the chest girth of the normal-type yak was generally better than that of the other types. Combining the data in Table 3, the normal types of individuals showed better growth traits. The results indicated that the *SOX6* gene variable region affects some important growth traits of yaks.

## 4. Discussion

CNV was originally applied for studying the rod chromosomes of *Drosophila* [30]. Subsequently, researchers have studied CNVs for exploring human genetic diseases and livestock breeding [31,32]. With the in-depth study of genomics, CNV has been at the center of much attention, and researchers have made significant progress in this field. Due to the crucial nature of the economic traits, more attention needs to be paid to breeding individuals of excellent characteristics using molecular breeding. So far, previous studies have revealed a correlation between the CNV and some traits of animals. CNV has been studied in livestock such as chickens [33], pigs [34], sheep [2], cattle [29], etc. In addition, there was a significant correlation between the *HPGDS*-CNV and body weight aged 12, 18, and 30 months Ashidan yaks, and the body length of 18, 30-month-old yaks has been illustrated in a previous study of our group. The *HPGDS* gene can be employed as a candidate gene for MAS in yak breeding [35].

*SOX6* was originally isolated from the testis of the adult mice [15,16], and was known to perform the function by immune-stimulating gene expression of the type II collagen and aggrecan during the proliferating phase [36]. Prior studies have noted the importance of this gene in invertebrate growth and development of invertebrates which can regulate mouse spermatogenesis and participate in controlling neural differentiation [37,38,39]. In addition, there were several reports that the *SOX6* gene was also closely related to muscle development, the gene expression was most abundant in the skeletal muscle of the adult mice [40,41,42,43,44]. Further research found that *SOX6* takes part in regulating muscle fiber types and skeletal muscle growth and development [45,46]. In summary, numerous previous studies described a connection between the *SOX6* gene and the type transition of the skeletal muscle fiber. Therefore, it is meaningful for us to explore the effects of *SOX6* on the growth traits in yaks.

Reviewing our study, the gene expression levels and *SOX6*-CNV were detected by qPCR. One interesting finding was that the *SOX6* gene was expressed in all seven tissues. Figure 1 shows that the *SOX6* gene was adequately expressed in the kidney and heart tissues. A possible explanation for this might be that the yaks possess a more efficient cardiopulmonary function and a metabolic system in the high altitude and low oxygen environment [47,48]. The higher expression level of the *SOX6* gene was found in the skeletal muscle tissues. Combined with the previously mentioned finding, the *SOX6* was found to be related to muscle development. A comparison of the findings with the expression profile of *SOX6* in the human tissues on NCBI confirmed the reliability of our data. Results on CNV of the *SOX6* gene displayed that *SOX6*-CNV was statistically significantly related to the withers height and the chest girth of the 6-month yaks, the withers height of the 18-month yaks, the chest girth of the 30-month yaks. These results might be explained by the fact that yaks need to adapt to the plateau environment. The yak calves were weaned until 6-month old, to ensure that they can get abundant nutrients from breast milk during lactation. The body composition changes corresponded during this period. To adapt to the plateau, if their muscles and fat were not fully developed, the yaks would adapt to the environment of low oxygen mainly by heart. The heart developed rapidly during this period, and the thoracic cavity gradually became larger and deeper. When the yaks were 18-mouths old, the development of the body was speculated to the abundant pasture during this period and the increasing feed intake of the yak. The nutrient intake was greatly helpful for the daily weight gain of the yak during the growth period, muscle and fat growth development [49]. In addition, the chest girth of 30-month-old yaks was found to be significantly correlated with *SOX6*-CNV, which might be the reason why *SOX6* gene expression in the chest muscle was higher than that in the leg muscle [50]. The above results indicated that the *SOX6* gene might affect the growth and development of yaks, which might be related to the unique adaptability of the yak to the plateau.

The development of animal husbandry in the plateau areas is dispensable for the yaks and is necessary for selecting and breeding yaks. However, the slow growth of yaks has been a major problem in yak breeding. The withers height and chest girth are important growth traits for improving the defect of yak performance and economic benefits, of course, this requires molecular breeding technology. This study has identified that the *SOX6*-CNV is associated with the phenotype of the yaks and has a significant correlation with the chest girth at 6-mouth age and 30-mouth age and withers height at 6-mouth age and 18-mouth age of the Ashidan yak. According to the experimental data, the CNV advantage types of chest girth and withers height were not consistent at all ages, but there were tendencies for the individuals with the normal type to have a higher value on the growth traits. In addition, it is not practical to breed the yak only at a certain age since it does not consider the whole growth period of the yaks. Comprehensively, in this study, the normal type of CNV should have better advantages. It is possible that the *SOX6* gene can be a reference gene for yak breeding in the future. The research can not only provide a reference for the genetic improvement of yaks but also complement the research deficiency of the *SOX6* gene in the growth traits of yaks. Moreover, further research on different yak breeds should be conducted, especially other hybrid improved breeds with outstanding growth performance. Moreover, the mechanism of the *SOX6*-CNV affecting the growth traits needs further investigation.

## 5. Conclusions

The research aimed to assess if the *SOX6* gene influences the growth traits of yaks. This study has examined the impact of the CNV of the *SOX6* gene in yaks, and the *SOX6*-CNV was found to be significantly correlated to the chest girth and the withers height of 6-month yak (*p* < 0.05), the withers height of 18-month yak (*p* < 0.05), and the chest girth of 30-month yak (*p* < 0.05). The expression of the *SOX6* gene in the yak tissues was detected at the mRNA level, and a high expression of the *SOX6* gene was found in the muscle and kidney tissues. The evidence from this study suggests that the *SOX6* gene might influence the growth and development of the yak muscles, which provided a reference for studying the *SOX6* gene and can be considered as a candidate gene by molecular technique to assist in yak breeding.

## Figures and Tables

**Table 1:** Main test reagent equipment and manufacturers.

Reagents and Instruments	Manufacturer
EasyPure Blood Genomic DNA Kit	TransGen Biotech, Beijing, China
Trizol reagent	TransGen Biotech, Beijing, China
PrimeScript™ Reagent Kit and gDNA Eraser	TaKaRa Bio Inc., Dalian, China
RNase-free DNase	TaKaRa Bio Inc., Dalian, China
Thermo Scientific NanoDrop 2000C	ThermoFisher Scientific Inc., Waltham, MA, USA
LightCycler^®^ 96 Instrument	Roche, Basel, Switzerland

**Table 2:** Details of primers.

Level	Gene	Primer Sequence (5′–3′)	Product Length (bp)	Tm (°C)
DNA	*SOX6*	F: GCAACTACCACACCGTCACCTC	114	59
		R: TCCGCCGTCTGTCTTCATACCA		
DNA	*BTF*	F: AACCAGGAGAAACTCGCCAA	166	59
		R: TTCGGTGAAATGCCCTCTCG		
mRNA	*SOX6*	F: CGTTTGGGCAGGAGTTTGGA	148	60
		R: CGTTTGGTGGCTGTGGAGTT		
mRNA	*β*-*Actin*	F: GCAGGTCATCACCATCGG	158	60
		R: CCGTGTTGGCGTAGAGGT		

F: forward primer; R: reverse primer.

**Table 3:** ANOVA of the correlation between *SOX6*-CNV and four growth traits in 311 Ashidan yaks.

Age	Growth Trait	CNV (Type Mean ± SE)			*p* Value
		Loss (n = 17)	Normal (n = 182)	Gain (n = 112)	
6 months	Body weight (kg)	83.82 ± 2.826	84.45 ± 0.762	83.89 ± 0.996	0.896
	Withers height (cm)	94.94 ± 1.133 ^a^	94.80 ± 0.410 ^ab^	93.19 ± 0.475 ^b^	0.036 *
	Body length (cm)	91.76 ± 1.868	91.61 ± 0.492	92.82 ± 0.422	0.829
	Chest girth (cm)	121.76 ± 2.006 ^b^	124.81 ± 0.569 ^a^	122.78 ± 0.731 ^b^	0.047 *
12 months	Body weight (kg)	83.35 ± 3.240	83.49 ± 0.778	81.13 ± 1.028	0.185
	Withers height (cm)	90.47 ± 1.275	90.77 ± 0.294	90.04 ± 0.408	0.348
	Body length (cm)	95.35 ± 1.228	96.27 ± 0.351	95.18 ± 0.459	0.156
	Chest girth (cm)	115.76 ± 1.675	117.64 ± 0.358	116.45 ± 0.511	0.090
18 months	Body weight (kg)	121.55 ± 4.857	123.67 ± 1.126	121.63 ± 1.308	0.487
	Withers height (cm)	99.50 ± 1.863 ^b^	101.03 ± 0.513 ^b^	102.77 ± 0.537 ^a^	0.038 *
	Body length (cm)	99.86 ± 1.522	102.32 ± 0.479	101.28 ± 0.519	0.160
	Chest girth (cm)	136.79 ± 2.358	137.56 ± 0.851	139.08 ± 0.850	0.434
30 months	Body weight (kg)	155.64 ± 3.827	155.50 ± 1.380	155.48 ± 1.625	0.999
	Withers height (cm)	100.20 ± 1.277	100.08 ± 0.451	98.86 ± 0.544	0.208
	Body length (cm)	113.87 ± 1.591	113.81 ± 0.477	112.53 ± 0.638	0.257
	Chest girth (cm)	147.20 ± 2.066 ^ab^	148.10 ± 0.722 ^a^	145.14 ± 0.953 ^b^	0.045 *

^a, b^ indicate significant differences (*p* < 0.05); * *p* < 0.05.

## Data Availability

The study did not report any data.

## Supplementary Materials

The following are available online at https://www.mdpi.com/article/10.3390/ani12223074/s1. Table S1: The homogeneity of variance test of each trait. Table S2: The test of normality of each trait.

## References

[1] Feuk L., Marshall C.R., Scherer W.S.W. (2006). Structural variants: Changing the landscape of chromosomes and design of disease studies. Hum. Mol. Genet..

[2] Shi S.Y., Li L.J., Zhang Z.J., Wang E.Y., Huang Y.Z. (2019). Copy number variation of MYLK4 gene and its growth traits of Capra hircus (goat). Anim. Biotechnol..

[3] Korbel J.O., Urban A.E., Affourtit J.P., Godwin B., Grubert F., Simons J.F., Kim P.M., Palejev D., Carriero N.J., Du L. (2007). Paired-End Mapping Reveals Extensive Structural Variation in the Human Genome. Science.

[4] Rogers R.L., Cridland J.M., Shao L., Hu T.T., Peter A., Thornton K.R. (2014). Landscape of standing variation for tandem duplications in Drosophila yakuba and Drosophila simulans. Mol. Biol. Evol..

[5] Stothard P., Choi J., Basu U., Sumnerthomson J.M., Yan M., Liao X., Moore S.S. (2011). Whole genome resequencing of Black Angus and Holstein cattle for SNP and CNV discovery. BMC Genom..

[6] Iourov I.Y., Vorsanova S.G., Yurov Y.B. (2019). The variome concept: Focus on CNVariome. Mol. Cytogenet..

[7] Dorshorst B., Molin A.M., Rubin C.J., Johansson A.M., StrMstedt L., Pham M.H., Chen C.F., Hallb F.K., Shwell C.A., Andersson L. (2011). A Complex Genomic Rearrangement Involving the Endothelin 3 Locus Causes Dermal Hyperpigmentation in the Chicken. PLoS Genet..

[8] Seo B.Y., Park E.W., Ahn S.J., Lee S.H., Jeon J.T. (2007). An accurate method for quantifying and analyzing copy number variation in porcine KIT by an oligonucleotide ligation assay. BMC Genet..

[9] Xiong Y.C., Cao X.K., He H., Song L.F., Huang C., Dang L.P., Zhang J.Y., Lei C.Z., Cheng H., Qi X.L. (2016). The Detection of GBP4 Gene Copy Number Variation and Its Effect on Five Bovine Growth Traits. China Cattle Sci..

[10] Wright D., Boije H., Meadows J.R.S., Bed’hom B., Gourichon D., Vieaud A., Tixier-Boichard M., Rubin C.J., Imsland F. (2009). Copy Number Variation in Intron 1 of *SOX5* Causes the Pea-comb Phenotype in Chickens. PLoS Genet..

[11] Ding L., Wang Y., Kreuzer M., Guo X., Mi J., Gou Y., Shang Z., Zhang Y., Zhou J., Wang H. (2013). Seasonal variations in the fatty acid profile of milk from yaks grazing on the Qinghai-Tibetan plateau. J. Dairy Res..

[12] Xu Y., Zhang L.Z., Shi T., Zhou Y., Cai H.F. (2013). Copy number variations of MICAL-L2 shaping gene expression contribute to different phenotypes of cattle. Mamm. Genome.

[13] Zhang G.M., Li Z., He H., Song C.C., Zhang Z.J. (2018). Associations of GBP2 gene copy number variations with growth traits and transcriptional expression in Chinese cattle. Gene.

[14] Henrique D., Junior G., Cesar A., Freua M.C., Gomes R.C., Da L., Leme P.R., Fukumasu H., Carvalho M.E., Ventura R.V. (2016). Copy number variations and genome-wide associations reveal putative genes and metabolic pathways involved with the feed conversion ratio in beef cattle. J. Appl. Genet..

[15] Kondoh H., Kamachi Y. (2009). *SOX*-partner code for cell specification: Regulatory target selection and underlying molecular mechanisms. Int. J. Biochem. Cell Biol..

[16] Liu C.F., Lefebvre V. (2015). The transcription factors *SOX9* and *SOX5*/*SOX6* cooperate genome-wide through super-enhancers to drive chondrogenesis. Nucleic Acids Res..

[17] Hamada-Kanazawa M., Ishikawa K., Ogawa D., Kanai M., Kawai Y., Narahara M., Miyake M. (2004). Suppression of *SOX6* in P19 cells leads to failure of neuronal differentiation by retinoic acid and induces retinoic acid-dependent apoptosis. FEBS Lett..

[18] Lefebvre V., Crombrugghe B.D., Behringer R.R. (2001). The transcription factors L-*SOX5* and *SOX6* are essential for cartilage formation. Dev. Cell.

[19] Lefebvre V. (2010). The *SOXD* transcription factors—*SOX5*, *SOX6*, and *SOX13*—Are key cell fate modulators. Int. J. Biochem. Cell Biol..

[20] Jackson H.E., Ono Y., Wang X., Elworthy S., Cunliffe V.T., Ingham P.W. (2015). The role of *SOX6* in zebrafish muscle fiber type specification. Skelet. Muscle.

[21] Sun D.M. (2009). Studies on the Transcriptional Regulation Mechanisms of Transcription Factors Required for Skeletal Development.

[22] Aza-Carmona M., Shears D.J., Yuste-Checa P., Barca-Tierno V., Hisado-Oliva A., Belinchón A., Benito-Sanz S., Rodríguez J.I., Argente J., Campos-Barros A. (2011). SHOX interacts with the chondrogenic transcription factors *SOX5* and *SOX6* to activate the aggrecan enhancer. Hum. Mol. Genet..

[23] Klewer H.S.E., Samson R.A. (2000). *SOX6* is a candidate gene for p100H myopathy, heart block, and sudden neonatal death. Proc. Natl. Acad. Sci. USA.

[24] Jia C., Wang H., Li C., Wu X., Yan P. (2019). Genome-wide detection of copy number variations in polled yak using the Illumina BovineHD BeadChip. BMC Genom..

[25] Gilbert R.P., Bailey D.R., Shannon N.H. (1993). Linear body measurements of cattle before and after 20 years of selection for postweaning gain when fed two different diets. J. Anim. Sci..

[26] Edwards E., Saunders N., Logan J., Sails A.D., Ad S. (2009). Real-Time PCR: Current Technology and Applications.

[27] Ali S., Srivastava A.K., Chopra R., Aggarwal S., Garg V.K., Bhattacharya S.N., Bamezai R. (2013). IL12B SNPs and copy number variation in IL23R gene associated with susceptibility to leprosy. J. Med. Genet..

[28] Bae J.S., Cheong H.S., Kim L.H., Namgung S., Park T.J., Chun J.Y., Kim J.Y., Pasaje C., Jin S.L., Shin H.D. (2010). Identification of copy number variations and common deletion polymorphisms in cattle. BMC Genom..

[29] Liu M., Li B., Shi T., Huang Y., Liu G.E., Lan X., Lei C., Chen H. (2019). Copy number variation of bovine SHH gene is associated with body conformation traits in Chinese beef cattle. J. Appl. Genet..

[30] Knudsen O. (1958). Studies on spermatocytogenesis in the bull. Int. J. Fertil..

[31] Yang X., Song Z., Wu C., Wang W., Li G., Zhang W., Wu L., Lu K. (2018). Constructing a database for the relations between CNV and human genetic diseases via systematic text mining. BMC Bioinform. [Electron. Resour.].

[32] Ma Y.L., Wen Y.F., Cao X.K., Cheng J., Chen H. (2019). Copy number variation (CNV) in the IGF1R gene across four cattle breeds and its association with economic traits. Arch. Anim. Breed..

[33] Jing Z., Wang X., Cheng Y., Wei C., Hou D., Li T., Li W., Han R., Li H., Sun G. (2020). Detection of CNV in the SH3RF2 gene and its effects on growth and carcass traits in chickens. BMC Genet..

[34] Wang L.G., Zhang Y.B., Yan H., Zhang L.C., Hou X.H., Liu X., Wang L.X. (2019). Identification of Candidate Genes for Porcine Bone Rate Traitsby Genome-wide Association of Copy Number Variation. Acta Vet. Zootech. Sin..

[35] Huang C., Ge F., Ren W., Zhang Y., Liang C. (2020). Copy Number Variation of the HPGDS Gene in the Ashidan yak and Its Associations with Growth Traits. Gene.

[36] Ikeda T., Kawaguchi H., Kamekura S., Kou I., Hoshi K., Nakamura K., Ikegawa S., Chung U. (2004). Combination of *SOX5*, *SOX6* and *SOX9* (the *SOX* trio) provides signals suffucient for introduction of permanent cartilage. Arthritis Rheum..

[37] Frances C., Edwina W., Paul D., Peter K., Alan A. (1995). The Sry-related HMG box-containing gene *SOX6* is expressed in the adult testis and developing nervous system of the mouse. Nucleic Acids Res..

[38] Cantone M., Küspert M., Reiprich S., Lai X., Eberhardt M., Gttle P., Beyer F., Azim K., Küry P., Wegner M. (2019). A gene regulatory architecture that controls region-independent dynamics of oligodendrocyte differentiation. Glia.

[39] Zhang L., Xue Z., Yan J., Wang J., Liu Q., Jiang H. (2019). LncRNA Riken-201 and Riken-203 modulates neural development by regulating the *SOX6* through sequestering miRNAs. Cell Prolif..

[40] Sluijter J., Mil A.V., Vliet P.V., Metz C., Liu J., Doeven Da Ns P.A., Goumans M.J. (2010). MicroRNA-1 and -499 regulate differentiation and proliferation in human-derived cardiomyocyte progenitor cells. Arter. Thromb. Vasc. Biol.

[41] Lübbert M., Jones P.A. (2014). Epigenetic Regulation of Globin Genes and Disturbances in Hemoglobinopathies.

[42] An C.I., Dong Y., Nobuko H. (2011). Genome-wide mapping of *SOX6* binding sites in skeletal muscle reveals both direct and indirect regulation of muscle terminal differentiation by *SOX6*. BMC Dev. Biol..

[43] Rooij E.V., Quiat D., Johnson B.A., Sutherland L.B., Qi X., Richardson J.A., Kelm R.J., Olson E.N. (2009). A family of microRNAs encoded by myosin genes governs myosin expression and muscle performance. Dev. Cell.

[44] Smits P. (2004). *SOX5* and *SOX6* are needed to develop and maintain source, columnar, and hypertrophic chondrocytes in the cartilage growth plate. J. Cell Biol..

[45] Quiat D., Voelker K.A., Pei J., Grishin N.V., Grange R.W., Bassel-Duby R., Olson E.N. (2011). Concerted regulation of myofiber-specific gene expression and muscle performance by the transcriptional repressor *SOX6*. Proc. Natl. Acad. Sci. USA.

[46] Wang X.Y. (2017). MicroRNA-499-5p Regulates the Formation of Porcine Slow Myofibers by Targeting *SOX6*. Master’s Thesis.

[47] Wiener G., Han J., Long R. (2011). The yak. Rap Publ..

[48] Qiu Q., Zhang G., Ma T., Qian W., Wang J. (2012). The yak genome and adaptation to life at high altitude. Nat. Genet..

[49] Sun P.F. (2021). Study on Main Factors Influencing the Growth of Yak under the Condition of Grazing Grass at Different Growing Times during Warm Season. China Dairy Cattle.

[50] Lin X.R. (2016). Copy Number Variation of the *SOX6* Affects Chicken Growth Traits. Master’s Thesis.

